# The genetics of hypertrophic cardiomyopathy

**DOI:** 10.21542/gcsp.2018.36

**Published:** 2018-08-12

**Authors:** Mohammed Akhtar, Perry Elliott

**Affiliations:** Bart’s Heart Centre, St Bartholomew’s Hospital, London & University College London

## Abstract

Hypertrophic cardiomyopathy (HCM) is most commonly transmitted as an autosomal dominant trait, caused by mutations in genes encoding cardiac sarcomere proteins^1–3^. Other inheritable causes of the disease include mutations in genes coding for proteins important in calcium handling or that form part of the cytoskeleton^4–6^. At present, the primary clinical role of genetic testing in HCM is to facilitate familial screening to allow the identification of individuals at risk of developing the disease^7,8^. It is also used to diagnose genocopies, such as lysosomal^9–11^ and glycogen storage disease which have different treatment strategies, rates of disease progression and prognosis^12–14^. The role of genetic testing in predicting prognosis is limited at present, but emerging data suggest that knowledge of the genetic basis of disease will assume an important role in disease stratification^15–17^ and offer potential targets for disease-modifying therapy in the near future^18^.

## Genetic architecture of HCM

**Figure 1.**
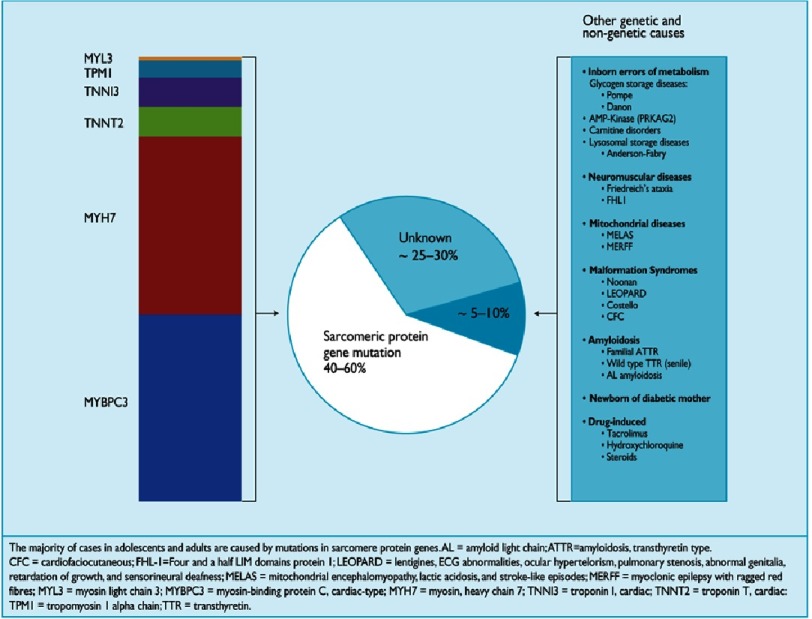
Representation of the percentage of hypertrophic cardiomyopathy cases accounted for by pathogenic mutations in sarcomeric and non-sarcomere genes. Elliott PM, et al; 2014 ESC Guidelines on diagnosis and management of hypertrophic cardiomyopathy: The Task Force for the Diagnosis and Management of Hypertrophic Cardiomyopathy of the European Society of Cardiology (ESC), European Heart Journal 2014; 35 (39): 2733–2779, doi:10.1093/eurheartj/ehu284. Reproduced by permission of Oxford University Press on behalf of the European Society of Cardiology. © European Society of Cardiology 2014. All rights reserved. For permissions please email journals.permissions@oup.com. This figure is not included under the Open Access license of this publication.

Familial HCM is characterized by locus and allelic heterogeneity, with a high frequency of novel individual mutations^7,19^. The first genetic mutation to be identified was a single base substitution in the *MYH7* gene encoding ß-myosin heavy chain, a key component of the cardiac sarcomere^20^. Since then, many different mutations in *MYH7* and other genes of the cardiac sarcomere have been identified. In 5–10% of cases, HCM is caused by mutations in genes that cause metabolic disorders^21–23^, neuromuscular disease^24–26^ or inherited genetic syndromes including Noonan syndrome^27–29^ (Figure 1).

## The cardiac sarcomere

**Figure 2.**
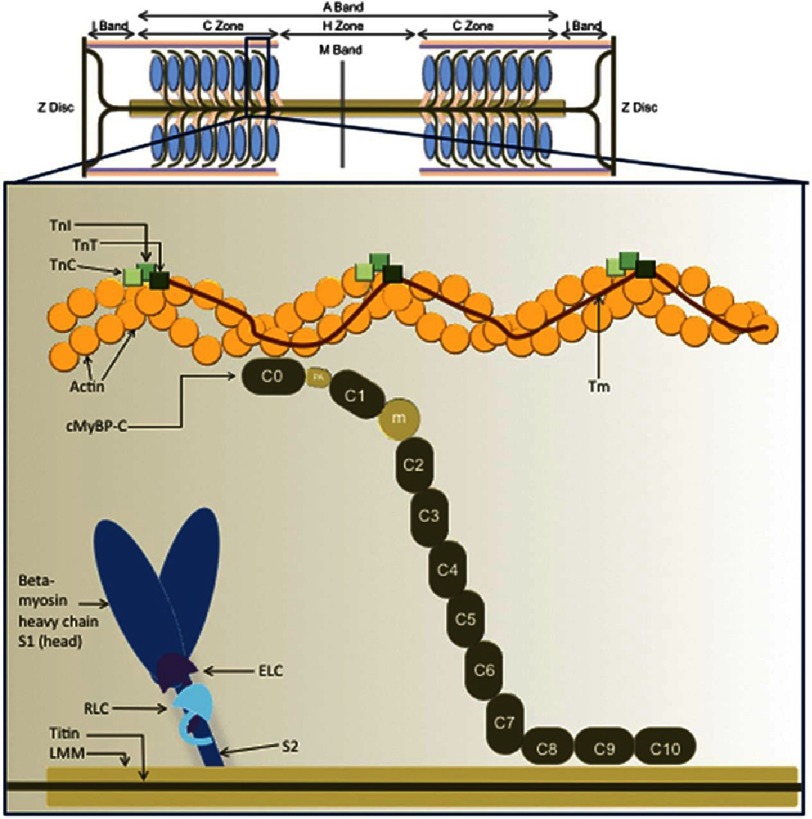
Representation of the cardiac sarcomere with associated proteins and interactions. Reproduced from Ref. [31]. Abbreviations: TnI, Troponin I; TnC, Troponin C; TnT, Troponin T; Tm, Tropomyosin; c-MYBP-C, cardiac myosin binding protein-c; ELC, Essential Light Chain; RLC, Regulatory Chain; LMM, Light meromyosin. The domains of cMyBP-C are numbered from C0–C10; m is the regulatory motif between domains C1 and C2 and contains the PKA phosphorylation site; PA represents a proline / alanine-rich linker sequence between domains C0 and C1.

Every cardiomyocyte is composed of myofibrils that run longitudinally along the cell and are transversely subdivided into contractile units called sarcomeres^30,31^. The sarcomere constitutes the fundamental motor unit of the cardiomyocyte and is composed of two principal components – the *thick filament* composed of around 300 molecules of myosin, each made up of 2 protein units of *β*- or *α*-myosin heavy chain and 4 myosin light chain molecules, and the *thin filament* composed of repeating actin molecules, closely associated with the regulatory troponin complex (troponin T (TnT), troponin I (TnI) and troponin C (TnC)) and *α*-tropomyosin^31–33^. An additional protein, cardiac myosin-binding protein C, contributes to the regulation of actin–myosin interaction and cross-bridge kinetics^31^ (Figure 2).

Muscle contraction results from the interaction between myosin and actin in which the globular head of the myosin molecule bends towards and then binds to actin, contracts, releases actin, and then initiates a new cycle. The links between the myosin head and actin are called *cross-bridges*; the contraction of the myosin S1 region which requires the hydrolysis of ATP to release energy is called the *power stroke*^31,34^.

Electrical activation of the heart and cardiomyocyte contraction are coupled through the intracellular movement of calcium. Depolarisation of the cardiomyocyte cell membrane activates the L-type voltage-dependent calcium channels in the T tubule causing an influx of calcium into the cell that triggers opening of ryanodine-receptor channels in the adjacent sarcoplasmic reticulum with a rapid increase in cytosolic calcium^31^. Calcium binds to TnC, inducing an allosteric conformational change in TnI and TnT that is transmitted to tropomyosin and exposes the myosin-binding sites of actin allowing the cross-bridges to form^33^. The myosin heavy chain head, with ADP and inorganic phosphate bound to its nucleotide-binding pocket, then interacts with the exposed actin-binding sites followed by the release of ADP and inorganic phosphate, which occurs simultaneously with the power stroke. ATP then binds to the nucleotide-binding pocket of the myosin heavy chain head, which detaches from actin and myosin then hydrolyses ATP into ADP and inorganic phosphate restarting the contraction cycle^31^.

## Sarcomere mutations in HCM

Mutations in *ßMYH7* and *MYBPC3* account for 60–70% of HCM patients with pathogenic variants^1,19,32,35^. Mutations in other sarcomere and associated protein genes are listed in Table 1. Mutations in *MYH7* are predominantly missense, with single nucleotide-base substitutions resulting in a non-synonymous single amino acid substitution^20,36^. In contrast, the majority of mutations in *MYBPC3* are nonsense mutations due to insertion/deletions, splice-site variants or frameshifts causing a premature stop codon that results in a truncated protein transcript^1,32,37^.

**Table 1:** List of genes in which pathogenic mutations are associated with hypertrophic cardiomyopathy. The chromosome location and the proportion of HCM cases attributed to mutations in these specific genes are included.

Protein	Gene	Chromosome location	Proportion of HCM caused by mutations
**SARCOMERIC PROTEINS**			
*B*-Myosin Heavy Chain 7	*MYH7*	14q12	40–44%
Myosin-Binding Protein C 3	*MYBPC3*	11p11	35–40%
Troponin T	*TNNT2*	1q32	5–15%
Troponin I	*TNNI3*	19q13	5%
Tropomyosin alpha-1 chain	*TPM1*	15q22	3%
Regulatory Myosin Light Chain	*MYL2*	12q24	1–2%
Essential Myosin Light Chain	*MYL3*	3p21	1%
Actin	*ACTC1*	15q14	1%
Troponin C	*TNNC1*	3p21	<1%
**Z-DISK PROTEINS**			
ZASP –LIM binding domain 3	*LBD3*	10q22	1–5%
Alpha-Actinin-2	*ACTN2*	1q42	<1%
Ankyrin repeat domain containing protein –1	*ANKRD1*	10q23	<1%
Muscle LIM Protein	*CSRP3*	11p15	<1%
Myozenin-2	*MYOZ2*	4q26	<1%
Telethonin	*TCAP*	17q12	<1%
Vinculin	*VCL*	10q22	<1%
Nexilin	*NEXN*	1p31	<1%
Filamin C	*FLNC*	7q32	<1%
**SARCOMERE-ASSOCIATED PROTEINS**			
Desmin	*DES*	2q35	<1%
Four and a Half Lim Domain Protein –1	*FHL-1*	Xq26	<1%
**CALCIUM-HANDLING PROTEINS**			
Phospholamban	*PLN*	6q22	<1%
Calreticulin 3	*CALR3*	19p13	<1%
Calsequestrin 2	*CASQ2*	1p13	<1%
Junctophilin 2	*JPH2*	20q13	<1%
**HCM PHENOCOPIES (METABOLIC & LYSOSOMAL STORAGE DISORDERS)**			
AMP-gamma2 subunit	*PRKAG2*	7q36	Together with other HCM phenocopies account for 5–10% of HCM cases
Glucosidase A (Pompe’s disease)	*GAA*	17q25	
Alpha-Galactosidase A (Anderson-Fabry Disease)	*GLA*	Xq22	
Lysosomal-associated membrane protein 2 (Danon’s Syndrome)	*LAMP2*	Xq24	
**HCM PHENOCOPIES (Ras-MAPK)**			
Noonan Syndrome	*KRAS*	12p12	Together with other HCM phenocopies account for 5–10% of HCM cases
	*SOS1*	2p22	
	*PTPN11*	12q24	
	*RAF1*	3p25	
LEOPARD syndrome	*PTPN11*	12q24	
	*RAF1*	3p25	
**HCM PHENOCOPY (NEUROMUSCULAR DISORDERS)**			
Friedreich’s Ataxia	GAA expansion in *FXN*	9q13	Together with other HCM phenocopies account for 5–10% of HCM cases

The majority of missense mutations are believed to have a dominant negative effect in which the mutant protein is incorporated into the sarcomere, but its interaction with the normal wild-type protein disrupts normal sarcomeric assembly and function (poison polypeptide hypothesis)^31,38^. Individual missense mutations may change an amino acid in a highly-conserved residue, alter important kinase domains (affecting ligand interaction) or change surface-exposed portion of a molecule altering protein-protein interaction. Missense mutations can also cause protein misfolding and accelerated degradation by ubiquitin-proteasomal surveillance pathways.

In truncating mutations, haploinsufficiency is thought to be the major disease mechanism^39,40^. The reported absence of detectable truncated myosin-binding protein C in western-blot analysis of myectomy specimens of patients with this mutation may be due to nonsense mediated mRNA decay of abnormal transcripts or ubiquitin-mediated proteasomal degradation of aberrant truncated protein^40–43^.

Allelic heterogeneity can be partly explained by the effect of different mutations on the structure and function of the complete peptide. *ß*-myosin heavy chain, for example, consists of a globular head, an *α*-helical rod and a hinge region. The globular head contains binding sites for ATPase and actin as well as interaction sites for regulatory and essential light chains in the head-rod region^31^. The majority of disease-causing *ß*-myosin heavy chain mutations are found in one of four locations: the actin binding site, the nucleotide binding pocket, the hinge region adjacent to the binding site for two reactive thiols and in the *α*-helix close to the essential light chain interaction site^44^. Thus, different effects on protein function might be expected depending on the position of the mutation.

It has been speculated that the HCM disease phenotype results from reduced contractile function caused by altered actin-myosin interactions, and consequent inappropriate compensatory hypertrophic remodelling^45,46^. However, some *MYH7* mutations are associated with increased cardiomyocyte mechanical contractile forces *in vitro* and show an increase in calcium sensitivity, leading to increases in tension generation and ATPase activity. Animal and cell studies have also confirmed altered calcium homeostasis as a key contributor to the pathophysiological processes that lead to the development of LV hypertrophy^47,48^.

Troponin T mutations account for less than 5% of all cases of HCM but often show a particular phenotype. *TNNT2* mouse models show varying degrees of myocyte disarray and fibrosis with minimal LVH, in common with *TNNT2* disease expression in humans^49,50^. Troponin-mutated mice exhibit severely impaired myocardial relaxation, independent of the degree of fibrosis, and consistent with the finding of increased calcium sensitivity^51–53^. Some studies suggest that some *TNNT2* mutations are associated with a high incidence of sudden death which may also relate to calcium loading in cardiomyocytes^54,55^.

Findings from murine models of HCM suggest that the increased contractility seen with some mutations is at the expense of inefficient ATP utilization. Furthermore, animal and human studies suggest that HCM is associated with depleted energy stores and abnormal ATP/ADP ratios^56–59^. Inefficient utilization of ATP is also seen in metabolic disorders or mitochondrial cytopathies, which can produce a pattern of LV hypertrophy similar to that in sarcomeric HCM.

Recently, mutations in genes encoding z-disc proteins including myozenin (*MYOZ2*), telethonin (*TCAP*), alpha-actinin-2 (*ACTN2*), muscle LIM protein (*CRP3*) and nexilin (*NEXN*) have been implicated in HCM^31^. Some mutations in Z-disc proteins have a pleotropic effect, causing an HCM phenotype in certain individuals and a DCM phenotype in others within the same family^60^.

## Childhood-onset HCM

The importance of sarcomeric protein gene mutations in childhood hypertrophic cardiomyopathy is unknown. The observation that the development of left ventricular hypertrophy in individuals with familial disease often occurs during the period of somatic growth in adolescence has led to the suggestion that sarcomeric protein disease in very young children is rare^61,62^. However, studies of children with HCM have shown that, as in adults, sarcomeric protein gene mutations account for approximately 50% of cases of idiopathic HCM, even in infants and young children^63,64^.

## Phenotypic variability

There is a substantial variation in the expression of identical mutations indicating that other genetic and possibly environmental factors influence disease expression. The effect of age is perhaps the best characterized factor, most patients developing ECG and echocardiographic manifestations of the disease after puberty and before the age of thirty^65,66^. Sex also appears to influence disease expression in sarcomere protein disease^67,68^. Other potential modifying factors include renin-angiotensin-aldosterone system gene polymorphism^69–71^, and the occurrence of homozygosity and compound heterozygosity^72–74^.

## Genetic advancements

The impact of more rapid, cost-effective gene sequencing methods, together with improved cellular models including induced pluripotent stem cells, gene-editing technology, larger sequenced control cohorts and deep clinical phenotyping in cases means that we are at an exciting crossroads in the genetics of HCM. This provides researchers with significant opportunity to identify novel candidate causative genes and to potentially allow clearer genotype-phenotype correlations to be made, thereby laying the foundation for more personalised patient care.
